# Fragment-pair based drug molecule solubility prediction through attention mechanism

**DOI:** 10.3389/fphar.2023.1255181

**Published:** 2023-10-10

**Authors:** Jianping Liu, Xiujuan Lei, Chunyan Ji, Yi Pan

**Affiliations:** ^1^ School of Computer Science, Shaanxi Normal University, Xi’an, China; ^2^ Computer Science Department, BNU-HKBU United International College, Zhuhai, China; ^3^ Faculty of Computer Science and Control Engineering, Shenzhen Institute of Advanced Technology, Chinese Academy of Sciences, Shenzhen, China; ^4^ Shenzhen Key Laboratory of Intelligent Bioinformatics, Shenzhen Institute of Advanced Technology, Shenzhen, China

**Keywords:** drug discovery, drug molecules, solubility prediction, attention mechanism, fragments

## Abstract

The purpose of drug discovery is to identify new drugs, and the solubility of drug molecules is an important physicochemical property in medicinal chemistry, that plays a crucial role in drug discovery. In solubility prediction, high-precision computational methods can significantly reduce the experimental costs and time associated with drug development. Therefore, artificial intelligence technologies have been widely used for solubility prediction. This study utilized the attention layer in mechanism in the deep learning model to consider the atomic-level features of the molecules, and used gated recurrent neural networks to aggregate vectors between layers. It also utilized molecular fragment technology to divide the complete molecule into pairs of fragments, extracted characteristics from each fragment pair, and finally fused the characteristics to predict the solubility of drug molecules. We compared and evaluated our method with five existing models using two performance evaluation indicators, demonstrating that our method has better performance and greater robustness.

## 1 Introduction

Drug R&D (Research and Development) is a complex process involving many disciplines and technological fields, mainly including medicine, chemistry, biology, data science (David et al., 2009; Fleming, 2018). Drug discovery is the first step in the entire drug R&D process, the goal of which is to find molecules that can treat a certain disease. This can be achieved through the acquisition of naturally existing or artificially synthesized compounds, virtual screening, high-throughput screening (Hughes et al., 2011; Brown and Boström, 2018). Drug discovery is a challenging task as it involves sifting through billions of molecules within a time span of approximately 12 years and a cost of 2 billion U.S. dollars until the drug is launched (Cai et al., 2020; Gupta et al., 2021). In the process of drug discovery, screening the properties of relevant drug molecules can quickly eliminate drug candidates that do not meet the expected properties, resulting in significant reduction of both time and financial resources. The properties of drug molecules refer to the chemical and physical characteristics of drug molecules, which can help scientists predict the absorption, distribution, metabolism, and excretion behavior of drugs in the body, and thus help drug R&D personnel formulate strategies and optimize drug design (Chuang et al., 2020). The physical and chemical properties of drug molecules including molecular weight, polarity, lipophilicity, solubility, and electrophilicity, can help drug R&D professionals better understand the behavior of drug molecules in living organisms, thus guiding drug R&D (Lu et al., 2023). Among them, the solubility of molecules is a critical indicator of the degree of solvent solubility, as it determines the ability of drug molecules to dissolve in living organisms, referring to the solubility of drug molecules in water or other solvents. Solubility can affect the pharmacokinetic performance of drugs in the body (Ran et al., 2002; Sorkun et al., 2019). Therefore, drug developers need to consider the solubility of drugs to control its effective dose in living organisms. Predicting the solubility of drug molecules in solvents can help drug R&D personnel better understand the dissolution and permeation characteristics of drugs and help drug design and optimization (Li and Zhao, 2007; Sorkun et al., 2019). Therefore, predicting the solubility of drug molecules is a very important step in the drug discovery process. Traditional methods for determining the solubility of drug molecules in solvents require chemical experiments in the laboratory. Although these methods are reliable and accurate, the time and cost of the experiments are difficult to control. In addition, the results obtained by traditional experimental methods may be affected by experimental conditions, precision, and equipment, thus lacking conclusive universality and generalizability (Williams, 2000; Ran and Yalkowsky, 2001; Huuskonen et al., 2008).

With the development of technology, an increasing number of researchers are inclined to use computational methods to predict the properties of existing drug molecules, especially the solubility of drug molecules (Lee et al., 2022). These methods include molecular dynamics (MD) simulation (Brooks, 1995; Hospital et al., 2015) and the techniques based on quantitative structure-activity relationship (QSAR) models (Wang et al., 2015; Neves et al., 2018; Chen et al., 2021). Molecular dynamics simulation-based approaches have considerable potential in the prediction of the physical and chemical properties of drug molecules in drug discovery. Klimovich et al. (2015) have provided a detailed account of the use of the free energy of drug molecule dynamics in the prediction of drug molecule properties. QSAR methods utilize the correlation between the physical and chemical properties and the structural characteristics of drug molecules and their biological activity to predict the solubility of drug molecules. These methods commonly encompass regression analysis, principal component analysis, maximum likelihood estimation, and genetic algorithm. Dudek et al. (2006) have presented the methods for constructing the three main components of QSAR models.

With the widespread application of artificial intelligence technology, an increasing number of machine learning and deep learning methods have been widely adopted in drug discovery property prediction and bioinformatics (Vamathevan et al., 2019; Zemouri et al., 2019; Lei et al., 2021; Pan et al., 2022; Han Chengshan, 2023). In addition, some attention-based methods have been widely applied in bioinformatics (Bian et al., 2021; Guo et al., 2022; Guo and Lei, 2022). Wu et al. (2022) replaced the gating network with the attention mechanism to capture dynamic task relations in the study of drug molecule solubility, and utilized local fine-tuning and consensus prediction to further improve the prediction performance of the model. Tang et al. (2020) proposed a self-attention-based message passing neural network to study the relationship between chemical properties and structures in a interpretable way during their research on drug molecule lipophilicity and solubility. Zhang et al. (2022) proposed a novel method based on cluster constraints to investigate the potential data characteristics of drug repositioning, and predicted new associations between existing drugs and diseases.

Graph convolutional neural network methods have also been further applied in the field of drug molecule property prediction (Zhao et al., 2021; Fang et al., 2022; Li et al., 2022). Zeng et al., (2022) used a variant of graph neural network that combined attention mechanism with graph neural network to capture drug molecule features and performed prediction of drug molecule properties. Zhang et al. (2021) used multi-scale attention networks to predict the properties of drug molecules, and their research showed that better results can be obtained when using image segments of drug molecules.

Therefore, the main research goal of this paper is to quickly predict the solubility of pending drugs in the drug screening stage, thereby shortening the time of drug screening and saving a lot of time for drug screening. Based on existing methods, we propose a model named MolSOL, which uses a fragment-based attention model framework to study the solubility of drug molecules. Firstly, we decompose the drug molecule based on the structure of functional groups, and subsequently divide the complete molecular structure into fragments as inputs to the model. Secondly, we use graph attention networks to extract and learn features from each drug molecule fragment. Thirdly, we integrate the learned features of molecular fragments and perform an analysis to predict the solubility of drug molecules. The key contributions of this article are as follows:

(1) A model called MolSOL is proposed to separate the complete molecule at a single bond, so as to form a pair of molecular fragments to extract the characteristics of the molecule separately.
(2) The graph attention network was used to extract the characteristics of molecular fragment pairs and learn.
(3) Compared with other advanced methods, better performance can be obtained in terms of solubility of drug molecules.

## 2 Materials and methods

### 2.1 Dataset and data process

#### 2.1.1 Dataset

The ESOL (Delaney) dataset (Delaney, 2004) is a widely used dataset in the field of computational chemistry and drug discovery. It was originally introduced by John Delaney in 2004 and has been extensively utilized for solubility prediction tasks. The dataset contains information on the solubility of various organic compounds as measured in water at room temperature. The dataset consists of a total of 1,128 compounds with their corresponding experimental solubility values. The solubility values are reported as logarithmic molar concentration (logS), ranging from −11.6 to 6.04. A negative logS value indicates low solubility, while a positive value indicates high solubility.

The compounds included in the ESOL dataset cover a wide range of organic chemistry, representing diverse chemical classes and functional groups. This diversity allows for a comprehensive analysis of the relationship between molecular structure and solubility. However, it is important to note that the ESOL dataset also has some limitations and potential biases. Firstly, the dataset is primarily focused on organic compounds and may not be representative of the solubility behavior of inorganic or organometallic compounds. Additionally, the dataset predominantly contains relatively small and drug-like molecules, which may limit its applicability for larger or structurally complex molecules. Furthermore, it is worth mentioning that the ESOL dataset is based on experimental measurements, which can be subject to errors and inconsistencies. There might be variations in the experimental methodologies used to measure solubility, leading to potential inaccuracies in the dataset. Also, the dataset might have some missing values or outliers, which could impact the predictive models trained on it.

In summary, the ESOL (Delaney) dataset provides valuable information on the solubility of a diverse range of organic compounds. D espite its limitations and potential biases, it remains a widely used benchmark dataset in solubility prediction tasks.

#### 2.1.2 Data process

**FIGURE 1:**
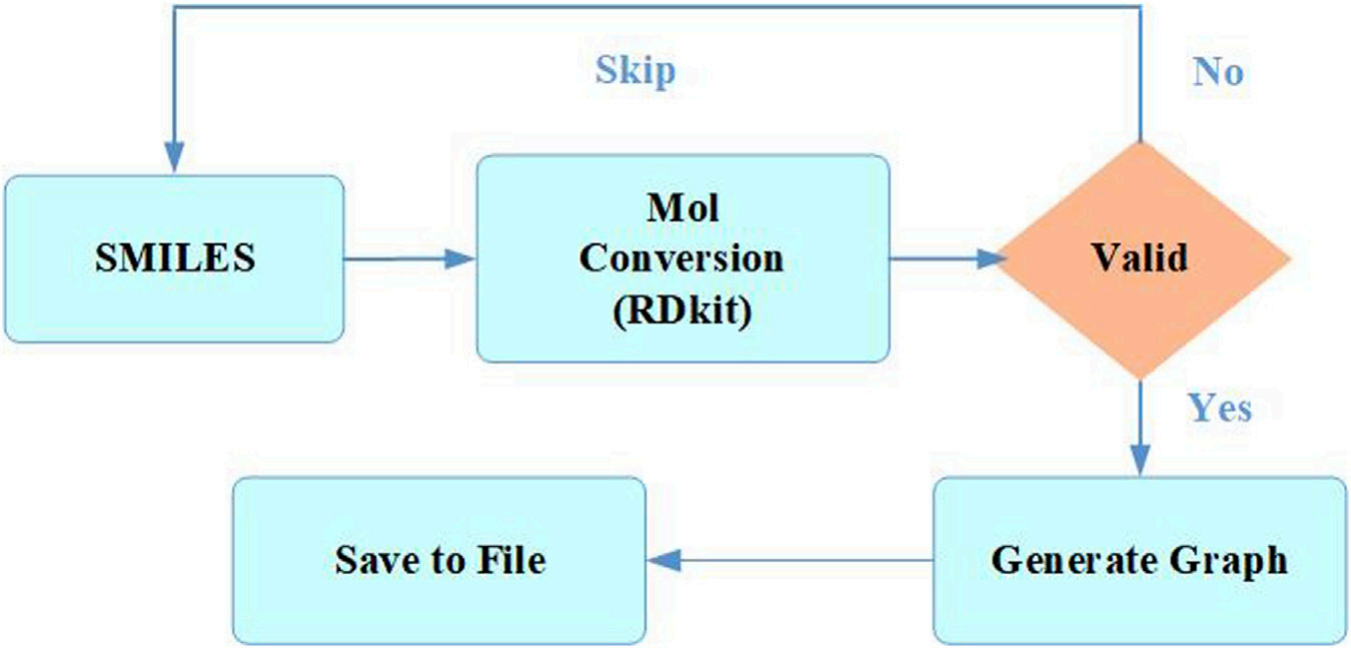
Preprocessing of molecules.

During the initial stage of data preprocessing, molecules with duplicate or missing information were removed to ensure that each chemical structure in the data was unique while maximizing the preservation of data properties (Yadav and Roychoudhury, 2018). Then, the Simplified Molecular Input Line Entry System (Weininger, 1988) (SMILES) data were processed one by one as shown in Figure 1. The SMILES were converted into a molecule using the Rdkit (Landrum, 2023) database to check its validity, and a molecular graph was generated using the node and edge features listed in Table 1 and being saved as input data for the model (Ahmad et al., 2023).

**TABLE 1:** Atom and bond features used for molecular graph construction.

Attribute	Size	Description
Atom symbol	15	[B, C, N, O, F, Si, P, S, Cl, As, Se, Br, Te, I, At]
Degree	8	Covalent bonds [0,1,2,3,4,5]
Charge	1	Electrical charge
Radical Electrons	1	radical electrons
Hybridization	6	[sp, sp^2^, sp^3^, sp^3^ d, sp^3^ d^2^, other]
Aromaticity	1	Atom is aromatic system part (true/false)
Hydrogens	5	Connected hydrogens [0,1,2,3,4]
Chirality	1	Atom chirality
Chirality type	2	R or S
Bond features Type	4	[single, double, triple, aromatic]
Conjugation	1	Bond conjugation
Ring	1	Bond is ring part
Stereo	4	[StereoZ, StereoNone, StereoE, StereoAny]

**FIGURE 2:**
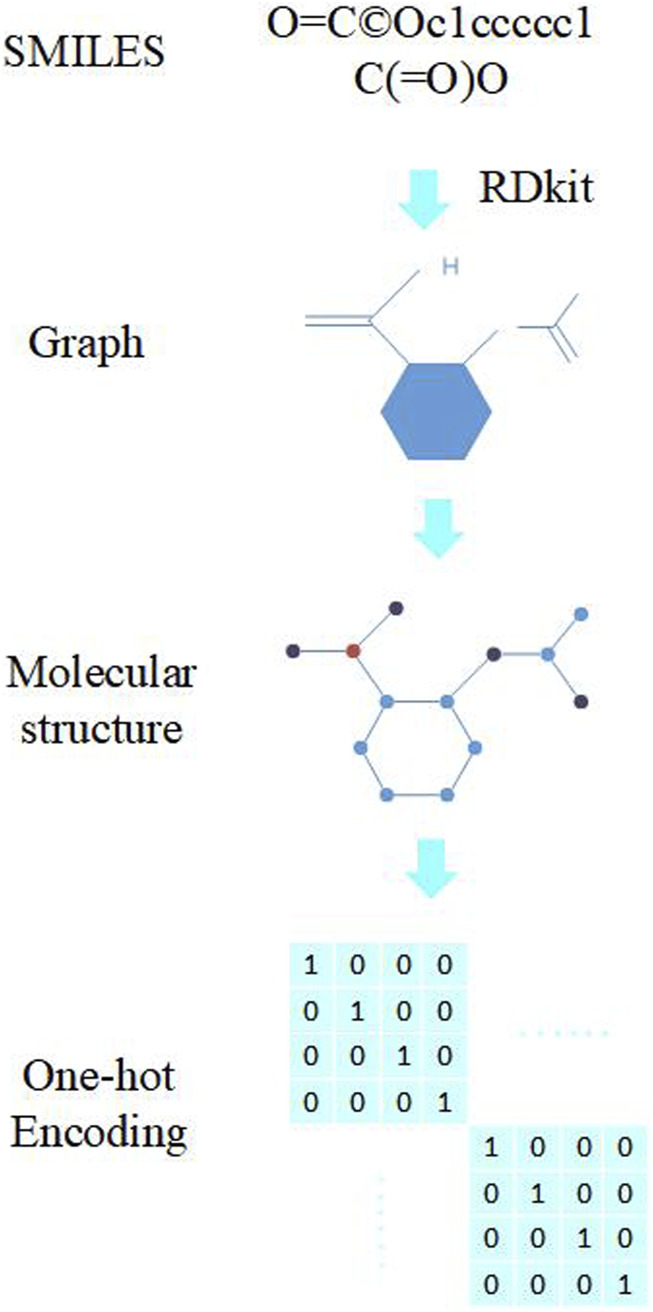
One-hot encoding.

The neural network in this paper requires inputs of molecules, which are represented using one-hot encoding. As shown in Figure 2, an entire aspirin molecule can be encoded using the adjacency matrix of its atoms. The adjacency matrix describes the relationship between atoms in the molecule, so the molecule can be decomposed into relatively independent nodes based on this relationship, with each node representing an atom. One-hot encoding is a commonly used representation method, which represents the feature vector of each atom as a long vector with only one position being 1 and other positions being 0. The position of the 1 represents the type of the atom, so we can use this method to express the features of the atom. By combining the one-hot vector of each atom with the adjacency matrix, we can obtain a complete molecular representation, which can be used as input to the neural network.

### 2.2 Molecular fragment

Molecular fragments refer to smaller subunits of molecular structures, usually composed of a few atoms or functional groups (Rees et al., 2004; Petros and Hajduk, 2009). Molecular fragments can be the basic skeleton of some compounds or small chemical molecules with specific functions. Compared to complete molecules, molecular fragments are more versatile and can be used in various fields, such as building structure-activity relationships, molecular design, and drug discovery. A common technique is to screen many small compounds (often molecular fragments) through high-throughput screening to evaluate their affinity for specific drug targets. Researchers can then combine these fragments to form larger compounds, where each molecular fragment can interact with the target protein. With such molecular design strategies, researchers can create millions of new compounds and evaluate their activity, thus discovering more effective drug candidates.

### 2.3 Molecular fragment extraction

A complete molecule can be divided into several different molecular fragments according to different rules. The most common methods for extracting molecular fragments include skeleton segmentation, substructure search, reaction division, and machine learning segmentation (Barnard, 1993; Duvenaud et al., 2015). Due to the complexity of the structure of organic substances, a complete molecule can be divided into multiple long skeletons and multiple molecular components. The number of molecular fragments may also increase with the number of acyclic single bonds since a molecule could contain a substantial quantity of such bonds.

**FIGURE 3:**
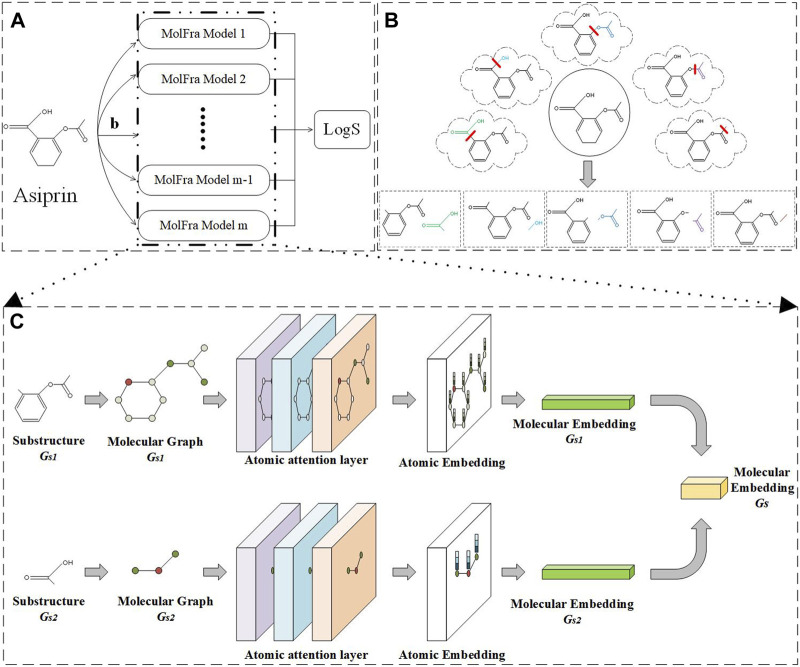
Overall model framework. **(A)** Method flow. **(B)** Molecular segmentation. **(C)** MolFra framework.

In this study, we utilize the fragility of single bonds in a molecule to mark all acyclic single bonds in the molecule as fragile bonds and extract molecular fragments accordingly. It should be noted that only one acyclic single bond is randomly broken during each extraction process in this study as shown Figure 3C, and thus, we generate two fragments as shown in Eq. 1:

$$G_{si}=Fragment\left(G_{s}\right),i=\left\{1,2\right\}$$ (1)

Here, *G*_*s*_ is a complete drug molecule and the *Fragment*() function is used to split the complete drug molecule. This approach can significantly reduce the computational cost and memory consumption of model training.

### 2.4 Methods

The model framework of the entire article is shown in Figure 3. Figure 3A shows the input and output of the entire network model. Firstly, a drug molecule graph *G*_*s*_ is the input, and the feature vector of the drug molecule is obtained through *m* MolFra models to predict the solubility of the drug molecule. Different possible molecular fragments of a drug molecule Asiprin resulting from the breakage of acyclic single bonds are shown in Figure 3B. The obtained drug molecule fragments are input into MolFra models in pairs, as shown in Figure 3C. The two molecular fragments *G*_*s1*_ and *G*_*s2*_ are separately input to the atomic attention network layers, obtaining the atomic embedding vectors for both molecular fragments. Finally, the atomic embedding vectors for the two molecular fragments are integrated into the molecular structure embedding vector and further integrated into a complete molecular embedding vector. Finally, the molecular average feature vector is obtained by taking the molecular embedding vectors for all possible pairs of molecular fragments and then a fully connected layer is used to predict the solubility of the drug molecule.

Xiong et al. (2020) proposed a graph convolutional neural network structure mainly based on graph attention networks to encode molecular structure information. In their paper, they indicated that their proposed graph neural network structure is significantly superior to existing works. This paper adopts this structured model as the molecular feature extraction network to obtain embedded vectors of the graph. The core of the graph attention mechanism is to obtain the context-aware vector of the target node by focusing on its neighboring and local environment.

### 2.5 Attention machine

The graph attention network structure in this article is shown in Figure 3C. Given two molecular fragment graphs *G*_*si*_ = {*V*_*si*_, *E*_*si*_, *Xi*_*atom*_, *Xi*_*bond*_}, where *i* = {1,2} represents molecular fragment 1 and molecular fragment 2, *V*_*si*_ = {*vi*_*1*_, *vi*_*2*_, …, *vi*_*N*_} represents the atoms in the *i-th* molecular fragment, *E*_*si*_ = {*ei*_*1*_, *ei*_*2*_, …, *ei*_*N*_} represents the bonds between two atoms in the *i-th* molecular fragment. *Xi*_*atom*_ = { $x_{1}^{atom}$ , $x_{2}^{atom}$ ,…, $x_{N}^{atom}$ }, *Xi*_*atom*_∈*R* ^*N×Fn*^ represents the feature matrix of chemical properties of atoms in the *i-th* molecular fragment, and *Xi*_*bond*_ = { $x_{1}^{bond}$ , $x_{2}^{bond}$ , …, $x_{N}^{bond}$ }, *Xi*_*bond*_∈*R* ^*N×Fe*^ represents the feature matrix of chemical properties of bonds in the *i-th* molecular fragment, where *F*_*n*_ and *F*_*e*_ respectively represent the dimensions of the chemical property vectors of atoms and bonds. All chemical properties in this article can be calculated using the RDkit library. The model proposed in this article is centered on the atom.

#### 2.5.1 Feature extraction

The attention network proposed in this article is referred as MolFra. Firstly, the two molecular fragments *G*_*s1*_ and *G*_*s2*_ are input into the network. The feature information is extracted using *l*_*1*_ and *l*_*2*_ layer attention networks to generate the atom node embedding vector features *H*_*1*_ = {*a*_*1*_, … , *a*_*N*_} and *H*_*2*_ = {*b*_*1*_, … , *b*_*N*_}, respectively, where *H*_*1*_ and *H2* belong to *R* ^*N×F*^, and *F* is the dimension of the embedding vector. In order to calculate the graph embedding, the two molecular fragments *G*_*s1*_ and *G*_*s2*_ are contracted into two graphs *s1* and *s2*. Two graphs are constructed respectively, denoted as $G_{s1}^{\prime }=\left\{{V1}_{s1}^{\prime },{E1}_{s1}^{\prime },{X1}_{Node}^{\prime }\right\}$ and $G_{s2}^{\prime }=\left\{{V2}_{s2}^{\prime },{E2}_{s2}^{\prime },{X2}_{Node}^{\prime }\right\}$ , where ${V1}_{s1}^{\prime }$ = {*s1*,*v1*_*1*_, … ,*v1*_*N*_} and ${V2}_{s2}^{\prime }$ = {*s2*,*v2*_*1*_, … ,*v2*_*N*_}, ${E1}_{s1}^{\prime }=\left\{e_{si},i\in V_{s1}\right\}$ and ${E2}_{s2}^{\prime }=\left\{e_{si},i\in V_{s2}\right\}$ . In the node feature matrix, ${X1}_{node}^{\prime }=\left\{{x1}_{s}^{\prime },{x1}_{1}^{\prime },\ldots ,{x1}_{N}^{\prime }\right\}$ and ${X2}_{node}^{\prime }=\left\{{x2}_{s}^{\prime },{x2}_{2}^{\prime },\ldots ,{x2}_{N}^{\prime }\right\}$ , where ${X1}_{node}^{\prime }$ , ${X2}_{node}^{\prime }$ ∈*R* ^*(N+1)×F*^. The feature vectors in the hypergraphs are initialized as follows:

$$x_{s1}^{\prime }=\frac{1}{N}\sum_{i\in V_{s1}}a_{i}$$ (2)

$$x_{s2}^{\prime }=\frac{1}{N}\sum_{i\in V_{s2}}b_{i}$$ (3)

$$x2_{i}^{\prime }=b_{i},i\in V_{s2}$$ (4)

$$x1_{i}^{\prime }=a_{i},i\in V_{s1}$$ (5)

Then, the node embedding vectors of graph *s1* and *s2* are extracted using *T*_*1*_ and *T*_*2*_ attention layers, respectively. These vectors then serve as the graph embeddings for the two molecular fragments.

#### 2.5.2 Attention layer

Each attention layer consists of two parts: aggregation and update. In the aggregation step, the target nodes *t*_*1*_ and *t*_*2*_ aggregate information propagated from their *1-hop* neighbors. The attention mechanism is used to assign weights to the messages for facilitating the model to aggregate important information. The aggregation steps of the attention mechanisms at layer *l*_*1*_ and *l*_*2*_ in the two hypergraphs can be formalized as follows:

$$\varepsilon_{t1i}^{l1}=leaky_{relu}\left(W_{1}\cdot \left[a_{t1}^{l1-1},a_{i}^{l1-1}\right]\right),i\in N\left(t1\right)$$ (6)

$$\varepsilon_{t2i}^{l2}=leaky_{relu}\left(W_{2}\cdot \left[a_{t2}^{l2-1},a_{i}^{l2-1}\right]\right),i\in N\left(t2\right)$$ (7)

$$C_{t1}^{l1}=elu\left(\sum_{i\in N\left(t1\right)}soft\max\left(\varepsilon_{t1i}^{l1}\right)W_{1}\cdot a_{i}^{l1-1}\right)$$ (8)

$$C_{t2}^{l2}=elu\left(\sum_{i\in N\left(t2\right)}soft\max\left(\varepsilon_{t2i}^{l2}\right)W_{2}\cdot a_{i}^{l2-1}\right)$$ (9)

The node embeddings of target node *t*_*1*_ and *t*_*2*_ and its *1-hop* neighbors are initialized as follows:

$$a_{t1}^{0}=x_{t1}^{atom}$$ (10)

$$b_{t2}^{0}=x_{t2}^{atom}$$ (11)

$$a_{i}^{0}=\left[x_{i}^{atom},x_{e_{ti}}^{bond}\right],i\in N\left(t1\right)$$ (12)

$$b_{i}^{0}=\left[x_{i}^{atom},x_{e_{ti}}^{bond}\right],i\in N\left(t2\right)$$ (13)

#### 2.5.3 Gated recurrent unit

**FIGURE 4:**
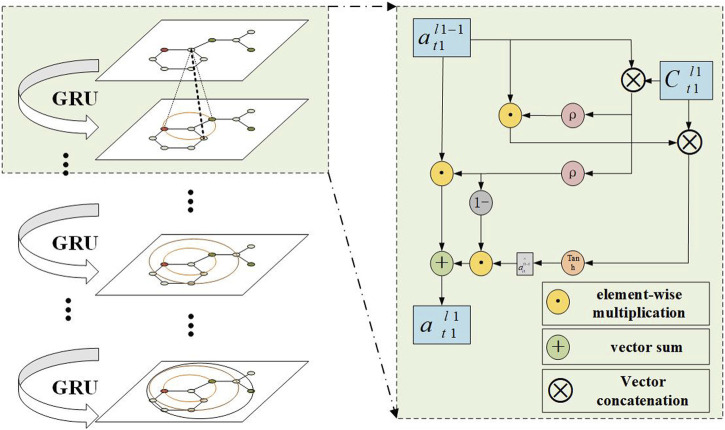
Aggregate embedded vector.

In the update step, a gated recurrent unit (GRU) was used as shown in Figure 4. It mainly accepts information aggregated from neighboring nodes and the embedded vector of the previous layer’s target node to generate the update state vector of the atom. This mechanism can be formally represented as:

$$a_{t1}^{l1}=GRU^{l1}\left(a_{t1}^{l1-1},C_{t1}^{l1}\right)$$ (14)

$$b_{t2}^{l2}=GRU^{l2}\left(a_{t2}^{l2-1},C_{t2}^{l2}\right)$$ (15)

#### 2.5.4 Integration of vectors

To obtain a global vector feature for a molecular fragment, we performed aggregation of atomic nodes and obtained the aggregated vector using Eqs 16, 17:

$$y_{1}^{1}=Aggregate\left(a_{i1}^{l1}\right),i=\left\{1,...,t\right\}$$ (16)

$$y_{1}^{2}=Aggregate\left(a_{i2}^{l2}\right),i=\left\{1,...,t\right\}$$ (17)

Here, $y_{1}^{1}$ represents the global vector feature of molecular fragment 1, while $y_{2}^{2}$ represents the global vector feature of molecular fragment 2. The *Aggregate*() function is used for aggregating the global features of molecular fragments. It merges the vector features of each fragment in a certain way to obtain the global vector feature representing the entire molecule.

By summing the global vector features of the two molecular fragments, a complete molecule vector representing the features of the entire molecule is obtained:

$${\overset{\wedge }{y}}_{1}=sum\left(y_{1}^{1}+y_{1}^{2}\right)$$ (18)

In this article, we first split a molecule into single bonds, then extract features and learn for each molecular fragment separately. Finally, we sum up the global feature vectors of the two molecular fragments. Since a molecule may have multiple single bonds, we also divide them into multiple pairs of molecular fragments. To obtain a complete drug molecule vector feature, we take the average of all the vector features after division as the final drug molecule feature vector, as shown in Eq. 19.

$$\overset{\wedge }{y}=mean\left(\overset{\wedge }{y_{i}}\right),i=1,2,...,m$$ (19)

Here, *m* represents the number of pairs of molecular fragments, and the *mean*() function is used to obtain the average value.

### 2.6 Optimization

In neural networks, loss functions are often used to calculate the difference between predicted and true values. As drug molecule solubility is a typical regression prediction task, we selected mean squared error (MSE), which calculates the square of the difference between the predicted and true values and then takes the mean. To optimize the performance and convergence speed of the model, we chose Adam (Adaptive Moment Estimation) for model optimization. Adam combines the advantages of Adagrad and RMSProp algorithms, enabling the adaptive adjustment of the learning rate of each parameter while considering the first and second moments of the gradient average during updates.

### 2.7 Complexity analysis

Our model is roughly divided into three parts: data preprocessing, graph neural network layers, and output layers. When calculating the complexity of the model, we mainly consider the complexity of these three parts.

In the data preprocessing part, the processing of chemical molecules is mainly performed, and these operations are linear. Therefore, O(n^2^ + m) can represent the complexity of this part, where O(n^2^) represents the complexity of modeling relationships between atoms, and O(m) represents the complexity of modeling chemical bond relationships.

After splitting the molecules into fragments, the model primarily processes the two network layers, *l*_*1*_ and *l*_*2*_. Therefore, we can understand that the model has *L* layers of networks, and the total complexity of the network layers in the model can be represented as O(L). The parameter L represents the number of network layers in your model. Each network layer is responsible for processing and transforming the input data. The choice of L depends on the complexity of the task at hand and the depth required to capture the necessary features and patterns in the data. Generally, having a deeper network (higher L) allows for more complex representations and potential improvements in performance. However, a very deep network can also lead to overfitting or slow convergence during training. Therefore, it is a trade-off that needs to be carefully considered and evaluated. Each network layer contains a certain number of neurons, and these neurons need to perform calculations and message passing. Therefore, the complexity of each layer of neurons in the model is represented as O(N), where *N* represents the number of neurons in each layer. The parameter N represents the number of neurons in each network layer. Neurons are the basic computational units that perform calculations and message passing in the model. The choice of N depends on the complexity of the input data and the capacity needed to capture the necessary information. Having a larger number of neurons (higher N) can potentially increase the model’s ability to learn complex patterns and representations. However, a larger model also requires more computational resources and can be more prone to overfitting if the dataset is not large enough to support the increased capacity. Thus, the complexity of the network layers in our model can be represented as O(L*N). The output layer is used for predicting the water solubility of the drug molecules and has relatively low complexity, which can be neglected.

Therefore, the overall complexity of our model can be represented as O(n^2^ + m + L*N).

## 3 Experiments and result

### 3.1 Metrics

To better evaluate the performance of the model, we selected two evaluation metrics commonly used by other researchers: mean absolute error (MAE) and root mean square error (RMSE). Here are the reasons for choosing MAE and RMSE:

1) Gap reduction: MAE and RMSE help quantify the prediction accuracy of a deep learning model by measuring the differences between actual observations and predicted values. This provides insights into the performance gap of the model.
2) Reflecting error magnitude: MAE and RMSE intuitively reflect the magnitude of prediction errors. MAE represents the average absolute difference between predicted values and true values, while RMSE represents the average squared difference. These metrics help assess the accuracy and stability of the model’s predictions.
3) Robustness to outliers: MAE and RMSE exhibit a certain level of robustness to outliers. Since RMSE involves squaring the differences, it is more sensitive to large errors and thus more influenced by outliers. On the other hand, MAE is smoother and less affected by outliers. This is particularly important in deep learning, as outliers can have a detrimental effect on the model.
4) Mathematical properties: Both MAE and RMSE have desirable mathematical properties, making them suitable for optimization and problem-solving. For instance, RMSE is differentiable, which is crucial for optimization algorithms like gradient descent used to adjust parameters in deep learning models.
5) Diversity and simplicity: MAE and RMSE are commonly-used evaluation metrics in deep learning, widely accepted and applicable in various scenarios. Moreover, they are relatively simple to calculate, allowing for easy comparison and performance assessment between models.

Overall, the advantages of MAE and RMSE as evaluation metrics in deep learning lie in their intuitiveness, robustness, and good mathematical properties. These metrics are widely used for model evaluation and optimization. However, it is important to choose the most appropriate evaluation metrics based on the specific problem and requirements. The calculation formulas for MAE and RMSE are as follows:

$$MAE=\frac{1}{n}\sum_{i=1}^{n}\left|\overset{\wedge }{y_{i}}-y_{i}\right|$$ (20)

$$RMSE=\sqrt{\frac{1}{n}{\sum_{i=1}^{n}\left|\overset{\wedge }{y_{i}}-y_{i}\right|}^{2}}$$ (21)

### 3.2 Parameter analysis

#### 3.2.1 Dataset ratio

**FIGURE 5:**
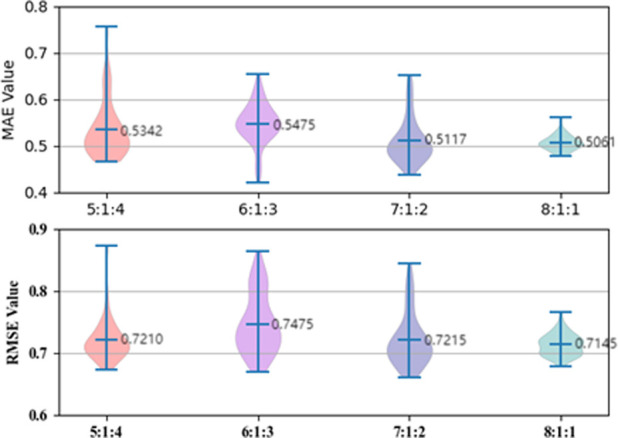
ESOL dataset ratio splitting.

Dividing the dataset into training, validation, and testing sets according to a certain proportion is usually an important operation in machine learning and deep learning tasks. Therefore, we first used the commonly used random splitting method, which divide the dataset into four ratios of 8:1:1, 7:1:2, 6:1:3, and 5:1:4 for training, validation, and testing sets, respectively, with a batch size of 200. The experimental results of the test set are shown in Figure 5, and we conclude that the 8:1:1 ratio provides the best model performance in terms of MAE and RMSE evaluation metrics. We believe that 8:1:1 ratio splitting provides enough training data, resulting in the best training effect of our model.

#### 3.2.2 Batch size

**FIGURE 6:**
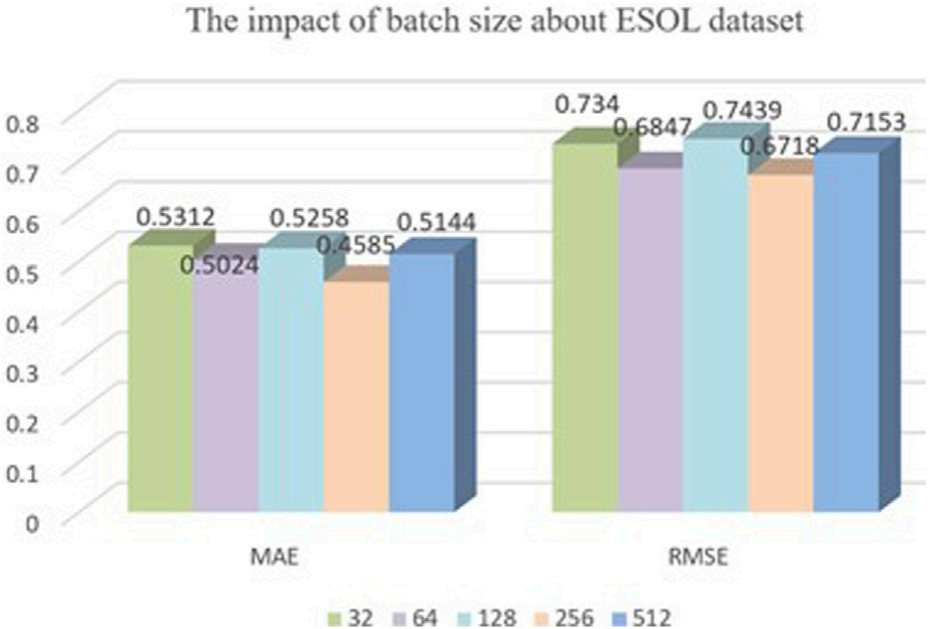
The impact of batch size about ESOL dataset.

Batch size is an important parameter that needs to be specified when training neural networks. It determines the number of samples that are fed into the model for training at each iteration. The choice of batch size has a significant impact on both the training efficiency and model performance. Therefore, it is vital to choose an appropriate batch size. In this model, we conducted experimental comparisons by selecting batch sizes of 32, 64, 128, 256, and 512 for training the model with a dataset ratio split of 8:1:1, as shown in Figure 6. From the graph, we can see that our model’s training performance is optimal when the batch size is 256, so we chose a batch size of 256.

#### 3.2.3 DataSet partitioning

There are two common forms of dataset partitioning: random partitioning and scaffold partitioning.

Random partitioning: The data is randomly divided into a training set, validation set, and test set. The advantage of random partitioning is that it can randomly select samples to make the distribution more reasonable and reduce bias. However, the disadvantage is that each run of the model will produce a different partition, which may affect the repeatability of the results.

Scaffold partitioning: The data is partitioned according to the original scaffold of the dataset. The advantage of skeleton partitioning is that the algorithm’s results are repeatable. The disadvantage is that it may introduce sample selection bias.

**FIGURE 7:**
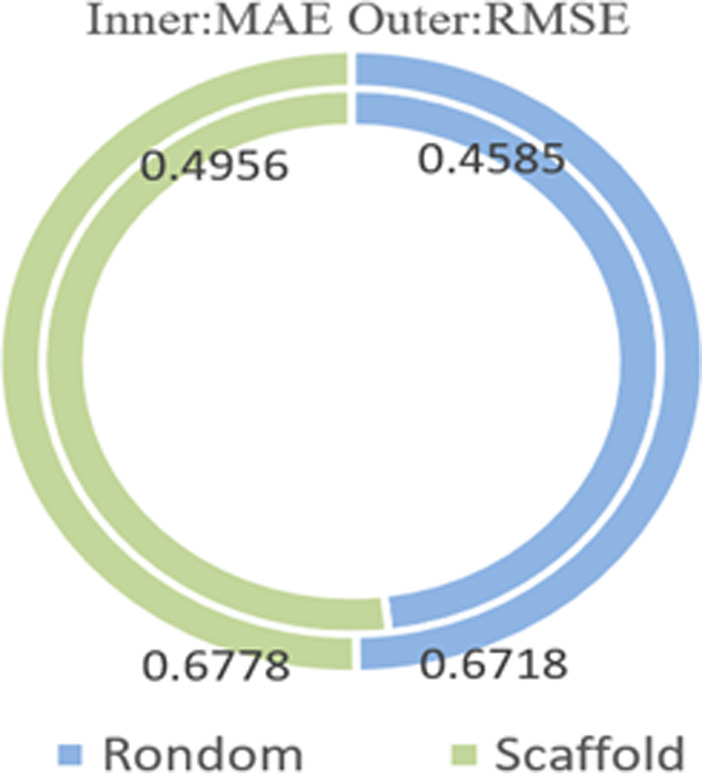
The impact of ESOL dataset partitioning.

We compared the random dataset splitting with scaffold dataset splitting under the condition of a batch size of 256 and the dataset ratio split of 8:1:1, as shown in Figure 7. The graph shows that the random splitting achieved better results with smaller values in three evaluation metrics than the scaffold splitting. We believe that this result is due to scaffold splitting handling more description and comparison of structural features between molecules, while random splitting is more suited for training and evaluating machine learning and deep learning models.

### 3.3 Experimental results on benchmarks

To better verify the performance of our model, our model compares the parameters of Table 2 with the five benchmark methods.

**TABLE 2:** The parameters used in model training.

Parameters	Value
Epoch	300
Batch Size	256
Dataset ratio split	8:1:1
Optimizer	Adam
Initial learning rate	0.0025
Atom layes	3
Mol layers	2
Weight_Decay	0.005
Drop rate	0.002
Early stop patience	40

Random forest is a supervised learning algorithm that generates a collection of decision trees through bootstrapping samples of compounds and features (Breiman, 2001). Random forest has the advantage of high scalability, can handle large-scale data sets, and can handle complex relationships in high latitude feature space, which makes it a powerful tool to predict drug molecular solubility, a problem with multiple characteristics. However, it requires a large amount of computing resources and memory to run, and the training process is often very time-consuming.

The MPN encoder is adapted from deep chemistry and chemical props and is implemented in Python, which is an open-source deep learning framework (Tang et al., 2020). MPN has the advantage of being able to capture local and global information, which helps to gain a more comprehensive understanding of the chemical information within molecules and improve the accuracy of water solubility prediction. However, compared to other traditional machine learning methods, it is more complex and requires more parameters and computational resources, which may result in higher computational costs and longer training time.

SAMPN is a message passing neural network model based on self-attention networks, which is adapted from the message passing neural network (Tang et al., 2020). SAMPN can directly learn the characteristics and structure of molecules without Feature engineering the molecules explicitly. This makes it suitable for dealing with various complex organic molecular structures and Chemical bond. But it usually requires a large amount of labeled data. This may be a challenge for certain specific fields or low abundance target attributes.

MultiMPN is a multi-task message passing neural network (Tang et al., 2020).

AlipSol is an attention-driven expert mixture model that explicitly reproduces the hierarchical structure of task relationships (Wu et al., 2022). AlipSol’s design can adapt to various types of molecular structures and chemical characteristics, and its prediction speed is usually fast, which can predict the water solubility of single or multiple molecules in a short time. This makes it a useful tool in fields such as high-throughput screening and virtual drug screening. But its performance depends on the quality and diversity of the dataset used for training. If the training set is insufficient or the sample distribution is uneven, it may affect the accuracy and applicability of the prediction results. Additionally, although AlipSol performs well in predicting molecular water solubility, it cannot cover all possible chemical structures and situations.

Table 3 shows the comparison results of our method and benchmark method on the test dataset, using a 8:1:1 random dataset partitioning method. It outperforms the benchmark method in all two performance evaluation metrics.

**TABLE 3:** Performance comparison of different methods of ESOL dataset.

	MAE	RMSE
RF	0.8011	1.1110
MPN	0.5126	0.7248
SAMPN	0.5046	0.7012
multiMPN	0.4737	0.6840
ALipSol	0.4615	0.6757
Ours	0.4585	0.6718

### 3.4 Ablation study

To verify the effectiveness of our model after splitting molecules into fragments, we also compared the performance on the complete molecules, as shown in Table 4. When we used molecule fragments, we obtained lower results in all three performance evaluation metrics, and the performance was also better. This demonstrates the effectiveness of our model after splitting molecules into fragments.

**TABLE 4:** Ablation experiment comparison about ESOL dataset.

	MAE	RMSE
Null-Fragment	0.4769	0.6865
Fragment	0.4585	0.6718

## 4 Case study

The main purpose of our study is to predict the water solubility of molecules in drug discovery and quickly screen out candidate drugs. We randomly selected six data from the dataset for result verification, as shown in Table 5. Among them, the three molecules C_6_H_12_NO_4_PS_2_, C_5_H_10_O, and C_5_H_10_O can spontaneously dissolve in water, and their predicted values are respectively different from the actual values by 0.004, 0.005, and 0.002. The three molecules C_3_H_8_O, C_5_H_5_N, and C_4_H_4_N_2_ cannot spontaneously dissolve in water and need to be added with additional energy to dissolve in water. The solubility of these three molecules are respectively different from the actual values by 0.004, 0.005, and 0.004. Based on the above six compounds, it is evident that our model’s predicted results are close to the actual values, serving as confirmation that our model is suitable for implementation in drug screening based on the prediction of molecular water solubility.

**TABLE 5:** Prediction of water solubility of ESOL.

SMILES	COP(=S) (OC)SCC(=O)N(C)C=O	CCCCC=O	CC(C)OC=O	CC(C)O	c1ccncc1	c1ccnnc1
Formula	C_6_H_12_NO_4_PS_2_	C_5_H_10_O	C_5_H_10_O	C_3_H_8_O	C_5_H_5_N	C_4_H_4_N_2_
Graph	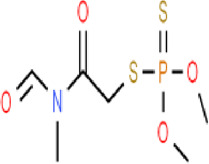	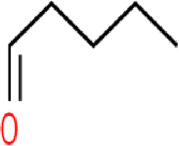	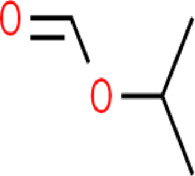	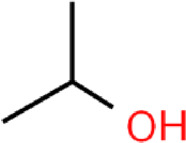	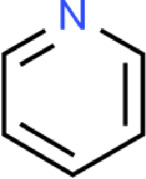	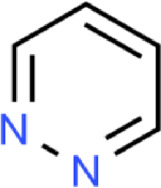
LogS	−1.995	−0.850	−0.630	0.430	0.760	1.100
Prediction	−1.991	−0.845	−0.628	0.426	0.755	1.096
SAMPN	−1.893	−0.843	−0.621	0.413	0.739	0.997
MPN	−1.801	−0.834	−0.602	0.401	0.723	0.961

To verify the generalizability of our model, we randomly selected six drug molecules from the AqSolDB (Wu et al., 2022) dataset to predict their water solubility, as shown in Table 6. The error between the actual values of the six drug molecules in the table and our predicted values ranges from 0.002 to 0.005, indicating that there is little difference in the actual error compared to the ESOL dataset. Therefore, it can be considered that our model has certain generalizability.

**TABLE 6:** Water solubility prediction on the AqSolDB.

SMILES	C[1]HCH=CHCH=CHCH=@1	C(C[2]CH=CHCH=CHCH=@2)#N	C(C[2]CH=CHCH=CHCH=@2) (=O)NH2	C(CH=CH2)#N	C(CH3) (=O)NH2	C(CH=CH2) (=O)NH2
LogS	−1.640	−1.000	−0.980	0.150	1.580	0.980
Prediction	−1.637	−0.996	−0.977	0.148	1.575	0.976

## 5 Conclusion

Solubility is of great importance in the physicochemical properties of drug molecules. In this study, we used drug molecule fragments and atom-level attention network techniques to predict the solubility of drug molecules. Our method was compared with five existing computational methods and achieved better results on all three performance evaluation metrics. This proves that the MolSol method proposed in our study can significantly reduce the prediction time for drug molecular water solubility, help screen and optimize candidate drugs, accelerate the development of new drugs, and increase the success rate. The model proposed in this article can predict the water solubility of drug molecules, and identify potential references for drug molecules considered ineffective or failed in other therapeutic areas.

In our future work, we will apply the MolSol model to the prediction of other drug properties to provide comprehensive support for drug design and development. We will also focus on combining water solubility prediction with other property prediction methods to construct a multi-property prediction model, providing comprehensive evaluation and guidance for drug development.

## Data Availability

The original contributions presented in the study are included in the article/Supplementary Material, further inquiries can be directed to the corresponding authors.

## References

[B1] Ahmad W. Tayara H. Chong K. T. (2023). Attention-based graph neural network for molecular solubility prediction. ACS Omega 8, 3236–3244. 10.1021/acsomega.2c06702 36713733 PMC9878542

[B2] Barnard J. M. (1993). Substructure searching methods: Old and new. J. Chem. Inf. Comput. Sci. 33, 532–538. 10.1021/ci00014a001

[B3] Bian C. Lei X.-J. Wu F.-X. (2021). Gatcda: Predicting circRNA-disease associations based on graph attention network. Cancers (Basel) 13, 2595. 10.3390/cancers13112595 34070678 PMC8198988

[B4] Breiman L. (2001). Random forests. Mach. Learn. 45, 5–32. 10.1023/A:1010933404324

[B5] Brooks C. L. (1995). Methodological advances in molecular dynamics simulations of biological systems. Curr. Opin. Struct. Biol. 5, 211–215. 10.1016/0959-440x(95)80078-6 7648323

[B6] Brown D. G. Boström J. (2018). Where do recent small molecule clinical development candidates come from? J. Med. Chem. 61, 9442–9468. 10.1021/acs.jmedchem.8b00675 29920198

[B7] Cai C. Wang S. Xu Y. Zhang W. Tang K. Ouyang Q. (2020). Transfer learning for drug discovery. J. Med. Chem. 63, 8683–8694. 10.1021/acs.jmedchem.9b02147 32672961

[B8] Chen L. Liu W.-G. Xiong F. Ma C. Sun C. Zhu Y.-R. (2021). 3D-QSAR, molecular docking and molecular dynamics simulations analyses of a series of heteroaryldihydropyrimidine derivatives as hepatitis B virus capsid assembly inhibitors. New J. Chem. 45, 22062–22076. 10.1039/D1NJ02542B

[B9] Chuang K. V. Gunsalus L. M. Keiser M. J. (2020). Learning molecular representations for medicinal chemistry. J. Med. Chem. 63, 8705–8722. 10.1021/acs.jmedchem.0c00385 32366098

[B10] David E. Tramontin T. Zemmel R. (2009). Pharmaceutical R&D: the road to positive returns. Nat. Rev. Drug Discov. 8, 609–610. 10.1038/nrd2948 19644471

[B11] Delaney J. (2004). Esol: Estimating aqueous solubility directly from molecular structure. J. Chem. Inf. Comput. Sci. 44, 1000–1005. 10.1021/ci034243x 15154768

[B12] Dudek A. Z. Arodz T. Gálvez J. (2006). Computational methods in developing quantitative structure-activity relationships (QSAR): a review. Comb. Chem. High. Throughput Screen 9, 213–228. 10.2174/138620706776055539 16533155

[B13] Duvenaud D. Maclaurin D. Aguilera-Iparraguirre J. Gómez-Bombarelli R. Hirzel T. Aspuru-Guzik A. (2015). Convolutional networks on graphs for learning molecular fingerprints. 10.48550/arXiv.1509.09292

[B14] Fang X. Liu L. Lei J. He D. Zhang S. Zhou J. (2022). ChemRL-GEM: Geometry enhanced molecular representation learning for property prediction. Nat. Mach. Intell. 4, 127–134. 10.1038/s42256-021-00438-4

[B15] Fleming N. (2018). How artificial intelligence is changing drug discovery. Nature 557, S55–S57. 10.1038/d41586-018-05267-x 29849160

[B16] Guo Y. Lei X. (2022). A pseudo-Siamese framework for circRNA-RBP binding sites prediction integrating BiLSTM and soft attention mechanism. Methods 207, 57–64. 10.1016/j.ymeth.2022.09.003 36113743

[B17] Guo Y. Lei X. Liu L. Pan Y. (2022). circ2CBA: prediction of circRNA-RBP binding sites combining deep learning and attention mechanism. Front. Comput. Sci. 17, 175904. 10.1007/s11704-022-2151-0

[B18] Gupta R. Srivastava D. Sahu M. Tiwari S. Ambasta R. K. Kumar P. (2021). Artificial intelligence to deep learning: machine intelligence approach for drug discovery. Mol. Divers 25, 1315–1360. 10.1007/s11030-021-10217-3 33844136 PMC8040371

[B19] Han Chengshan L. H. (2023). Research on coupling technology of multi-source heterogeneous information channels based on knowledge graph. J. Integration Technol. 12, 48–60. 10.12146/j.issn.2095-3135.20221026001

[B20] Hospital A. Goñi J. R. Orozco M. Gelpí J. L. (2015). Molecular dynamics simulations: advances and applications. Adv. Appl. Bioinform Chem. 8, 37–47. 10.2147/AABC.S70333 26604800 PMC4655909

[B21] Hughes J. P. Rees S. Kalindjian S. B. Philpott K. L. (2011). Principles of early drug discovery. Br. J. Pharmacol. 162, 1239–1249. 10.1111/j.1476-5381.2010.01127.x 21091654 PMC3058157

[B22] Huuskonen J. Livingstone D. J. Manallack D. T. (2008). Prediction of drug solubility from molecular structure using a drug-like training set. Sar. QSAR Environ. Res. 19, 191–212. 10.1080/10629360802083855 18484495

[B23] Klimovich P. V. Shirts M. R. Mobley D. L. (2015). Guidelines for the analysis of free energy calculations. J. computer-aided Mol. Des. 29, 397–411. 10.1007/s10822-015-9840-9 PMC4420631 25808134

[B24] Landrum G. (2023). RDKit: Open-source cheminformatics. Available at: https://zenodo.org/record/10398#.Ywl3uXFByUk (Accessed August 27, 2022).

[B25] Lee S. Lee M. Gyak K.-W. Kim S. D. Kim M.-J. Min K. (2022). Novel solubility prediction models: Molecular fingerprints and physicochemical features vs graph convolutional neural networks. ACS Omega 7, 12268–12277. 10.1021/acsomega.2c00697 35449985 PMC9016862

[B26] Lei X. Mudiyanselage T. B. Zhang Y. Bian C. Lan W. Yu N. (2021). A comprehensive survey on computational methods of non-coding RNA and disease association prediction. Brief. Bioinform 22, bbaa350. 10.1093/bib/bbaa350 33341893

[B27] Li J. Zhang C. Li Z. Nie R. Han P. Yang W. (2022). Gcmcdti: Graph convolutional autoencoder framework for predicting drug-target interactions based on matrix completion. J. Bioinform Comput. Biol. 20, 2250023. 10.1142/S0219720022500238 36350601

[B28] Li P. Zhao L. (2007). Developing early formulations: Practice and perspective. Int. J. Pharm. 341, 1–19. 10.1016/j.ijpharm.2007.05.049 17658228

[B29] Lu M. Yin J. Zhu Q. Lin G. Mou M. Liu F. (2023). Artificial intelligence in pharmaceutical sciences. Engineering. 10.1016/j.eng.2023.01.014

[B30] Neves B. J. Braga R. C. Melo-Filho C. C. Moreira-Filho J. T. Muratov E. N. Andrade C. H. (2018). QSAR-based virtual screening: Advances and applications in drug discovery. Front. Pharmacol. 9, 1275. 10.3389/fphar.2018.01275 30524275 PMC6262347

[B31] Pan Y. Lei X. Zhang Y. (2022). Association predictions of genomics, proteinomics, transcriptomics, microbiome, metabolomics, pathomics, radiomics, drug, symptoms, environment factor, and disease networks: A comprehensive approach. Med. Res. Rev. 42, 441–461. 10.1002/med.21847 34346083

[B32] Petros A. Hajduk P. J. (2009). Fragment-based drug discovery: A practical approach. J. Am. Chem. Soc. 131, 6036. 10.1021/ja902461y

[B33] Ran Y. He Y. Yang G. Johnson J. L. H. Yalkowsky S. H. (2002). Estimation of aqueous solubility of organic compounds by using the general solubility equation. Chemosphere 48, 487–509. 10.1016/S0045-6535(02)00118-2 12146628

[B34] Ran Y. Yalkowsky S. H. (2001). Prediction of drug solubility by the general solubility equation (GSE). J. Chem. Inf. Comput. Sci. 41, 354–357. 10.1021/ci000338c 11277722

[B35] Rees D. C. Congreve M. Murray C. W. Carr R. (2004). Fragment-based lead discovery. Nat. Rev. Drug Discov. 3, 660–672. 10.1038/nrd1467 15286733

[B36] Sorkun M. C. Khetan A. Er S. (2019). AqSolDB, a curated reference set of aqueous solubility and 2D descriptors for a diverse set of compounds. Sci. Data 6, 143. 10.1038/s41597-019-0151-1 31395888 PMC6687799

[B37] Tang B. Kramer S. T. Fang M. Qiu Y. Wu Z. Xu D. (2020). A self-attention based message passing neural network for predicting molecular lipophilicity and aqueous solubility. J. Cheminform 12, 15. 10.1186/s13321-020-0414-z 33431047 PMC7035778

[B38] Vamathevan J. Clark D. Czodrowski P. Dunham I. Ferran E. Lee G. (2019). Applications of machine learning in drug discovery and development. Nat. Rev. Drug Discov. 18, 463–477. 10.1038/s41573-019-0024-5 30976107 PMC6552674

[B39] Wang T. Wu M.-B. Lin J.-P. Yang L.-R. (2015). Quantitative structure-activity relationship: Promising advances in drug discovery platforms. Expert Opin. Drug Discov. 10, 1283–1300. 10.1517/17460441.2015.1083006 26358617

[B40] Weininger D. (1988). SMILES, a chemical language and information system. 1. Introduction to methodology and encoding rules. J. Chem. Inf. Comput. Sci. 28, 31–36. 10.1021/ci00057a005

[B41] Williams O. (2000). Solubility and Solubilization in Aqueous Media By Samuel H. Yalkowsky (University of Arizona). Oxford University Press: New York. 1999. xvi + 464 pp. $165. ISBN 0-8412-3576-7. J. Am. Chem. Soc. 122, 9882. 10.1021/ja0047424

[B42] Wu J. Wang J. Wu Z. Zhang S. Deng Y. Kang Y. (2022). ALipSol: An Attention-Driven Mixture-of-Experts Model for Lipophilicity and Solubility Prediction. J. Chem. Inf. Model. 62, 5975–5987. 10.1021/acs.jcim.2c01290 36417544

[B43] Xiong Z. Wang D. Liu X. Zhong F. Wan X. Li X. (2020). Pushing the Boundaries of Molecular Representation for Drug Discovery with the Graph Attention Mechanism. J. Med. Chem. 63, 8749–8760. 10.1021/acs.jmedchem.9b00959 31408336

[B44] Yadav M. L. Roychoudhury B. (2018). Handling missing values: A study of popular imputation packages in R. Knowledge-Based Syst. 160, 104–118. 10.1016/j.knosys.2018.06.012

[B45] Zemouri R. Zerhouni N. Racoceanu D. (2019). Deep Learning in the Biomedical Applications: Recent and Future Status. Appl. Sci. 9, 1526. 10.3390/app9081526

[B46] Zeng X. Xiang H. Yu L. Wang J. Li K. Nussinov R. (2022). Accurate prediction of molecular properties and drug targets using a self-supervised image representation learning framework. Nat. Mach. Intell. 4, 1004–1016. 10.1038/s42256-022-00557-6

[B47] Zhang Y. Lei X. Pan Y. Wu F.-X. (2022). Drug Repositioning with GraphSAGE and Clustering Constraints Based on Drug and Disease Networks. Front. Pharmacol. 13, 872785. 10.3389/fphar.2022.872785 35620297 PMC9127467

[B48] Zhang Z. Guan J. Zhou S. (2021). FraGAT: a fragment-oriented multi-scale graph attention model for molecular property prediction. Bioinformatics 37, 2981–2987. 10.1093/bioinformatics/btab195 33769437 PMC8479684

[B49] Zhao T. Hu Y. Valsdottir L. R. Zang T. Peng J. (2021). Identifying drug-target interactions based on graph convolutional network and deep neural network. Brief. Bioinform 22, 2141–2150. 10.1093/bib/bbaa044 32367110

